# A Case of O1 Vibrio Cholera Bacteremia and Primary Peritonitis in a Patient With Liver Cirrhosis

**DOI:** 10.4021/gr2009.12.1331

**Published:** 2009-11-20

**Authors:** Hussain Issa, Mahmoud Shorman, Bahaa Bseiso, Ahmed H. Al-Salem

**Affiliations:** a Department of Internal Medicine, King Fahad Specialist Hospital, Dammam, Saudi Arabia

**Keywords:** Vibrio cholera, Bacteremia, Peritonitis

## Abstract

Vibrio cholerae are Gram-negative bacteria that are differentiated into O1/O139 and non-O1/non-O139 serogroups depending on their ability to agglutinate with specific antiserum. In contrast to non-O1/non-0139 Vibrio cholerae, which are more prone to invade the bloodstream, Vibrio cholerae O1 is rarely the cause of bacteremia. We report a case of O1 Vibrio cholera bacteremia and primary peritonitis in a patient with liver cirrhosis. The literature on the subject is also reviewed.

## Introduction

Vibrio cholera is a facultative, gram negative anaerobic organism. It is classified on the basis of the somatic "O" surface antigen and based on this, they are divided into O1/O139 and non-O1/non-O139 serogroups [1]. Vibrio cholerae O1 is generally regarded as a non-invasive enterotoxigenic organism causing gastroenteritis of various severities [1]. This is in contrast to Vibrio cholera non-O1, although biochemically indistinguishable from Vibrio cholera O1, has been often associated with extra intestinal infection including septicemia, wound infection, peritonitis, skin infection, cellulitis, necrotizing fasciitis, endophthalmitis, ear infection, cholecystitis and meningitis [2-11]. This is usually seen in patients with diabetes mellitus, liver cirrhosis, and chronic renal failure on dialysis, hematological diseases, immunocompromised patients and post splenectomy [2, 6]. These infections are often associated with ingestion of contaminated seafood and associated with a high mortality rate [2, 6, 7, 11]. We report one unusual and rare case of bacteremia and primary peritonitis caused by O1 Vibrio cholera serotype Ojawa in a patient with liver cirrhosis.

## Case Report

A 47-year-old male presented to our emergency room with severe hematemesis and melena of two days duration and dizziness on the day of presentation. He is a known diabetic and hypertensive for the last 10 years and receiving insulin, atenolol and aspirin. He gave history of taking non-steroidal anti-inflammatory drugs for one week prior to presentation following tooth extraction. There was no history of previous similar attacks, no history of blood transfusion, alcohol intake or drug abuse. There was no history of diarrhea or intake of raw fish. Clinically, he was sick looking, pale, hypotensive and tachycardic. His temperature was 37.5°C, pulse 120/minute and blood pressure was 90/70 mmHg with postural drop. His chest was clear and had normal heart sounds. Abdominal examination revealed distension with ascites, epigastric tenderness and splenomegaly. There was no lower limb edema. His initial investigations showed a hemoglobin of 7.1 g/dl, white cell count o 19.29 x 10^3^/mm^3^ and platelets of 158,000/mm^3^. His total bilirubin was 29.9 mmol/L, direct bilirubin of 9.92 mmol/L, ALT (alanine transaminase) was 266 u/dl, and AST (aspartate transaminase) was 335 u/dl. His BUN, creatinine and electrolytes were normal. His PTT (Partial Thromboplastin Time) was normal while his PT (Prothrombin Time) was 17.52 seconds (normal: 10.4 - 14 seconds) and his INR (International Normalize Ratio) was 1.52 (normal: 0.8 - 1.2). He was admitted to the intensive care unit and started on intra venous octreotide, pantoprazol, intravenous fluids and blood transfusion. His blood pressure dropped further and he was electively intubated, ventilated and started on nor-epinephrine and intravenous antibiotics (Piperacillin and Tazobactam). Tapping of the ascetic fluid was done and this was diagnostic of spontaneous bacterial peritonitis (white blood cell count of 2,982/mm^3^, polymorph neutrophils were 92% and red blood cells of 618/mm^3^). He had upper gastrointestinal endoscopy which showed grade III esophageal varices with red color sign for which band ligation was performed. There were also fundal gastric varices, gastropathy and duodenopathy. His preliminary blood culture showed gram negative bacilli. He improved gradually, extubated and then shifted to the ward. He was investigated for chronic liver disease which showed negative hepatitis B and C. His abdominal ultrasound showed cirrhotic liver with signs of portal hypertension and splenomegaly but no focal liver lesion. Schistosomiasis screening was positive with a titer of 1:256, for which he was given praziquantel (40 mg/kg). The ascetic fluid culture showed no growth but the blood culture final result grew Vibrio cholera O1 serotype Ojawa sensitive to ampicilin, ciprofloxacin, bactrim, ceftazidine and tetracycline. He was started on levofloxacin in addition to Tazocin. His stool culture was negative. His repeat blood cultures came also to be negative. The initial blood culture was sent to another hospital for reconfirmation and the result came to be positive for Vibrio cholera O1 serotype Ojawa. The patient improved markedly and was discharged home in a good general condition to be followed-up for liver transplant workup.

## Discussion

Vibrio cholera is classified according to the somatic "O" surface antigen. Those that agglutinate with "O" antiserum are called O1 which are responsible for all epidemics of cholera. All other types that do not agglutinate with "O" antiserum are collectively called non-O1 Vibrio cholera [1]. The Vibrio cholera O1 group exists as two biotypes, classic and El Tor and both of them contain two major serotypes, the Ogawa and Inaba. An additional serotype, Hikojima was also described but this is rare. A new serotype was also discovered causing an epidemic in Bangladesh and India which was given the designation O139 Bengal after the area where it was first discovered. Since the discovery of this serotype, Vibrio cholera is classified into O1 and O139 and all other serogroups as non-O1/non-O139 [1].

Non-O1/non-O139 Vibrio cholerae are distributed worldwide. They are concentrated in fish and shelfish and contaminated undercooked seafood. Non O-1 Vibrio cholera infection is often associated with ingestion of contaminated seafood and its common presentation is sporadic gastroenteritis. They can however cause serious life threatening infections following the consumption of raw seafood or exposure of damaged skin to contaminated salt water. This is seen in patients with predisposing illnesses such as liver cirrhosis, chronic renal failure on dialysis, immunosuppresed patients, hematological diseases, diabetes mellitus and postsplenectomy [2, 6]. A mortality as high as 50% has been reported in affected patients [12]. A variety of infections caused by non-O1/non-O139 serotypes in such patients have been reported. These include septicemia, bacteremia, peritonitis, cellulitis, necrotizing fasciitis, skin infection, endophthalmitis, cholecystitis, meningitis and ear infection [2-11]. Lin et al reported a large series of 21 patients with liver cirrhosis who had non-O1 Vibrio cholera bacteremia and presented with ascites (95.2%), fever (81%), abdominal pain (52.4%), diarrhea (33.3%), and cellulitis with bullae formation (19%). In 10 of them there was concurrent spontaneous bacterial peritonitis [2]. The overall case-fatality rate was 23.8%, but 75% of the deaths were observed in patients with skin manifestation. Thamlikitkul on the other hand reported a 50% mortality among 20 patients with Vibrio cholera bacteremia, the majority of them had liver cirrhosis [12]. In 23 previously reported cases, the fatality rate was found to be 61.5% [11]. Ko et al reported 30 episodes of non-O1, non-O139 Vibrio cholerae infections [6]. They found three major clinical presentations: bacteremia with concurrent spontaneous bacterial peritonitis or invasive soft-tissue infections that occurred solely in cirrhotic patients; self-limited acute febrile gastroenteritis that occurred in patients with no underlying medical disease; and necrotizing fasciitis or cellulitis that often resulted from a wound on extremities. Other manifestations in their series included fatal pneumonitis in a drowned man and acute pyosalpinx [6].

The majority of the reported cases of non-O1/non-O139 Vibrio cholera infections were in patients with liver cirrhosis and taking the high mortality in consideration, patients with liver disease should be warned about the potential dangers of consuming raw or undercooked seafood as well as avoiding exposure of wounds to seawater. It is also important for physicians to consider this diagnosis in patients who have underlying risk factors specially where there is history regarding recent seafood ingestion.

Vibrio cholerae serogroup O1 is generally regarded as a non-invasive enterotoxigenic organism causing gastroenteritis of various severities. In contrast to non-O1/non-O139 Vibrio cholerae, which can invading the bloodstream causing bacteremia and septicemia, Vibrio cholerae O1 rarely causes bacteremia or invasive extraintestinal disease. Restrepo et al reported two patients with hepatitis C liver cirrhosis who developed bacteremia due to non-O1 Vibrio cholera in one and O1 serotype in the other [13]. They postulate that the hemolytic properties of these organisms contributed to their virulence in immunocompromised hosts. Thamlikitkul in 1990 reported 26 isolates of Vibrio cholera from the blood of patients, mostly adults with liver cirrhosis [12]. Only 20 of them were available for clinical analysis. The isolates in these patients were classified as non-O1 in 13 patients, 3 were Vibrio vulnificus and 10 were other Vibrio species. There were no details regarding the underlying diseases in these 10 patients. To our knowledge, this case report represents the second documented case of Vibrio cholera type O1 to cause bacteremia with sponateneous primary bacterial peritonitis in a patient with liver cirrhosis. The mode of infection in our patient is not clear as there was no history of documented contacts or recent traveling outside and Vibrio cholera is not endemic in Saudi Arabia. Our case shows that not only Vibrio cholera serotype non-O1/non-139 can cause bacteremia and primary peritonitis in patients with liver cirrhosis but also serotype O1. This must be kept in mind and a high index of suspicion is important in this regard as early diagnosis and administration of antibiotics may be curative.
